# Conserving Madagascar’s Amphibians and Reptiles Requires Collaboration between Scientists

**DOI:** 10.3390/ani14142091

**Published:** 2024-07-17

**Authors:** Franco Andreone, Angelica Crottini, Andolalao Rakotoarison, Fandresena Rakotoarimalala

**Affiliations:** 1 Museo Regionale di Scienze Naturali, Via G. Giolitti, 36, I-10123 Torino, Italy; 2 CIBIO, Centro de Investigação em Biodiversidade e Recursos Genéticos, InBIO Laboratório Associado, Campus de Vairão, Universidade do Porto, 4485-661 Vairão, Portugal; tiliquait@yahoo.it; 3 Departamento de Biologia, Faculdade de Ciências, Universidade do Porto, 4169-007 Porto, Portugal; 4 BIOPOLIS Program in Genomics, Biodiversity and Land Planning, CIBIO, Campus de Vairão, 4485-661 Vairão, Portugal; 5 Mention Environnement, Université de l’Itasy, Faliarivo Ambohidanerana, Soavinandriana Itasy 118, Madagascar; andomailaka@gmail.com; 6 School for International Training, Antananarivo 101, Madagascar; 7 Mention Zoologie et Biodiversité Animale, Faculté des Sciences, Université d’Antananarivo, Antananarivo 101, Madagascar; fandresenarak@gmail.com

Madagascar is well known for its exceptional biodiversity and striking endemicity levels, which are accompanied by high rates of deforestation and habitat alteration [1,2]. In recent decades, it has been possible to witness a progressive shrinking of Malagasy forests, which makes it more urgent than ever to improve our knowledge of this unique biota [3]. 

Among its peculiar fauna, amphibians and reptiles undoubtedly play an important ecological role and represent very speciose vertebrate groups: at the time of writing (May 2024), 414 species of amphibians (100% endemic [4]) and 437 species of reptiles (98% endemic) are known, and each year numerous species are described [5]. This situation highlights the urgent need to continue to document and describe the incredible biota of Madagascar and to promote its conservation. To discuss these threats and develop a strategy to protect the amphibians of Madagascar, two “A Conservation Strategy for the Amphibians of Madagascar” (ACSAM) meetings, an initiative coordinated by the regional IUCN/SSC Amphibian Specialist Group—Madagascar, were organized (the first in 2006 in Antananarivo and the second in 2014 in Ranomafana) [6,7]. These initiatives were mostly dedicated to the knowledge and protection of the highly diverse amphibian communities of Madagascar and the safeguard of their ecosystems, providing suggestions for needed actions to contribute to their conservation. More recently, an International Chameleon Day [8] was also launched and highlighted the peculiar role played by these animals as reptilian ambassadors [9]. 

Conservation in Madagascar, a country with clear economic difficulties, can realistically be achieved through a continuative synergy between knowledge, education, and people’s awareness. The development of a tight collaboration between local and foreign researchers and institutions has contributed to the development of a rich production of scientific contributions on the amphibians and reptiles of Madagascar [10,11]. Several publications are now produced by a variegated mix of local and foreign contributors, with national researchers frequently taking the lead, e.g., [12]. This is a much welcome development, followed by an increase in the number of scientific papers that include only Malagasy researchers. These outputs are often the result of fundamental research but play a significant role in informing decision-makers and stakeholders to facilitate the identification of more efficient conservation actions.

For these reasons, we are delighted to present the current Special Issue of Animals dedicated to advances in diversity, conservation, and taxonomy of Madagascar’s amphibians and reptiles. We hope that this issue, which has benefitted from a total waiver of publication fees and is published in open access, will represent a valuable contribution to the development of scientific knowledge about Madagascar’s biodiversity. In this issue, two papers have been produced by a team composed of only Malagasy researchers [13,14] and three contributions by a mix of local and foreign researchers [15,16,17]. 

The topics treated in this collection are sufficiently varied; more specifically, four papers deal with taxonomy and species distribution [13,16,17,18], and six with the ecology and conservation of Malagasy herpetofauna [14,15,19,20,21,22]. This repartition and the high number of discovered and described species that accumulated over the last three decades highlight how studies of geographic and taxonomic diversity remain a priority and should be considered a “must” to continually improve the comprehension of Madagascar’s herpetofauna [5]. 

We are glad to have succeeded in putting together this collection of papers where taxonomy, ecology, and biogeography approaches will hopefully be able to contribute to inform amphibian and reptile conservation. Studies on the ecology and behavior of Malagasy amphibians and reptiles are becoming increasingly available [23,24,25] and represent a significant contribution to the understanding of the life histories of these animals. The increase in attention toward these facets of the biology of amphibians and reptiles is particularly beneficial and remains an important aspect worth further investigation. 

Lastly, the impact of amphibian and reptile trade, as shown by Carpenter and Andreone [26], is a relevant contribution to an aspect that cannot be neglected, i.e., the necessary bridge between ecology and economy in a developing country, as also evidenced by a recent editorial [27]. An approach that considers conservation, economy, and local development remains crucial for the sustainable management of Malagasy natural resources.
